# Identifying Patients with Group 3 Pulmonary Hypertension Associated with COPD or ILD Using an Administrative Claims Database

**DOI:** 10.1007/s00408-022-00521-6

**Published:** 2022-03-29

**Authors:** Gustavo A. Heresi, Bonnie B. Dean, Howard Castillo, Henry F. Lee, Peter Classi, Dana Stafkey-Mailey, Alexander Kantorovich, Kellie Morland, Margaret R. Sketch, Benjamin S. Wu, Christopher S. King

**Affiliations:** 1 Department of Pulmonary and Critical Care Medicine, Respiratory Institute, Cleveland Clinic, Cleveland, OH US; 2 Xcenda, LLC, 5025 Plano Pkwy, Carrollton, TX 75010 US; 3 United Therapeutics Corporation, Research Triangle Park, Durham, NC US; 4 Advanced Lung Disease and Lung Transplant Clinic, Inova Fairfax Hospital, Falls Church, VA US

**Keywords:** Pulmonary hypertension, Real-world evidence, Retrospective claims studies, Algorithm

## Abstract

**Background:**

Group 3 pulmonary hypertension (PH) describes a subpopulation of patients with PH due to chronic lung disease and/or hypoxia, with chronic obstructive pulmonary disease (COPD) and interstitial lung disease (ILD) being two large subgroups. Claims database studies provide insights into the real-world treatment patterns and outcomes among these patients. However, claims data do not provide sufficient detail to assign the clinical subtype of PH required for identifying these patients.

**Methods:**

A panel of PH clinical experts and researchers was convened to discuss methodologies to identify patients with Group 3 PH associated with COPD or ILD in retrospective claims databases. To inform the discussion, a literature review was conducted to identify claims-based studies of Group 3 PH associated with COPD or ILD published from 2010 through June 2020.

**Results:**

Targeted title and abstract review identified 11 claims-based studies and two conference abstracts (eight based in the United States [US] and five conducted outside the US) that met search criteria. Based on insights from the panel and literature review, the following components were detailed across studies in the identification of Group 3 PH associated with COPD and ILD: (a) COPD or ILD identification, (b) PH identification, (c) defining the sequence between COPD/ILD and PH, and (d) other PH Group and Group 3 PH exclusions.

**Conclusion:**

This article provides recommended approaches and considerations for identifying and studying patients with Group 3 PH associated with COPD or ILD using administrative claims data that provide the foundation for future validation studies.

**Supplementary Information:**

The online version contains supplementary material available at 10.1007/s00408-022-00521-6.

## Background

Pulmonary hypertension (PH) is a condition characterized by elevation in pulmonary artery pressures of varying etiologies, which may result in substantial morbidity. PH is clinically classified into five groups, defined by the World Symposium on PH, according to differing pathological findings between groups, including underlying cause of disease, clinical presentation, and hemodynamic characteristics [1]. PH clinical classifications are also used by healthcare providers for medical management and by the Food and Drug Administration (FDA) for labeling of new drugs approved for the treatment of PH [1, 2].

Group 3 PH describes a subpopulation of patients with PH due to chronic lung disease and/or hypoxia. In particular, chronic obstructive pulmonary disease (COPD) and interstitial lung disease (ILD) are two large subgroups of chronic lung disease patients who often develop PH [2, 3]. In COPD, the prevalence rate ranges from 30 to 70% [4]. Because ILD is composed of multiple lung diseases, its prevalence is difficult to estimate. In idiopathic pulmonary fibrosis (IPF), the most common type of ILD, prevalence rates of PH range from 8 to 15% at initial diagnosis to 46% at evaluation for lung transplant and 86% at the time of transplant [5–10]. Wide ranges in prevalence can be due to heterogeneity in the definitions for PH, diagnostic modalities for PH, differences in patient populations, physiologic characteristics, and severity of underlying lung disease [4, 11].

Administrative claims are generated following healthcare utilization for the purposes of payment. Medical documentation is converted to standardized codes using uniform coding systems. The International Classification of Diseases (ICD), a medical classification system, is the international standard for reporting diseases and health conditions. In the US, the ICD, 9th Revision, Clinical Modification (ICD-9-CM) and ICD, 10th Revision, Clinical Modification (ICD-10-CM) provide a system of diagnostic codes assigned for each encounter.

ICD-9-CM diagnostic codes provide the level of detail to indicate PH but are not specific to groups of PH defined by World Symposium on PH. All groups of PH due to lung disease are generally coded under the same four-digit codes. The newer ICD-10-CM, implemented in October 2017, provides five-digit codes for PH with greater differentiation but was not required for billing and reimbursement until October 2018 [12]. The designated code for Group 3 PH is I27.23, and it remains to be seen how frequently and accurately the utilization of this code will be.

While collected for billing purposes, claims data can provide real-world evidence outside the setting of a clinical trial about the treatment patterns, risk factors, patient outcomes, healthcare resource utilization, and costs for patients covered by health insurance plans. However, using diagnostic codes in claims databases for the identification of patients with Group 3 PH associated with lung disease conditions like COPD and ILD is not sufficient. Additional considerations must be applied to patient identification to reduce misclassification and improve the usefulness of claims data to understand the medical management and health-related outcomes of patients with these conditions.

Recently, a focused review provided recommended algorithms for the identification of patients with pulmonary arterial hypertension (Group 1 PAH) [13]. Others have explored the use of machine-learning approaches to reduce selection bias in patient identification [14]. Our goal is to provide readers with information to determine the most appropriate methodology for claims-based patient selection under different types of research questions about Group 3 PH associated with COPD or ILD.

## Methods

A panel of US-based healthcare providers and researchers with expertise in PH was convened to discuss methodologies to identify patients with Group 3 PH associated with COPD or ILD in retrospective claims databases. Panel members included US-based practicing pulmonologists (*n* = 2), a nurse (*n* = 1), pharmacists (*n* = 6) with expertise in PH and backgrounds in public health and/or claims-based analysis, and researchers (*n* = 2) with expertise in pharmacoeconomics, epidemiology, and claims-based analysis.

We conducted a literature review to identify studies and explore considerations when using claims-based data to identify patients with Group 3 PH associated with COPD or ILD. The recommendations in this article are those of the authors convened for the discussion and are based on group consensus.

The literature review utilized EMBASE and MEDLINE (via EMBASE) to identify English language articles published from 2010 through June 2020, on adult patient population, including both US and international studies. We looked for claims-based studies, retrospective studies, or healthcare management-related studies, focused on both PH and lung diseases causing Group 3 PH, or that specifically mentioned Group 3 PH. The search terms for lung diseases and PH were derived from several sources [3, 15] and decided upon by the panel. Search terms were required in the title or abstract (Supplemental Table 1). We also referred to a similar effort around conceptualization of Group 1 PAH in order to provide further support [13].

Additionally, we reviewed abstracts from 2018 through 2020 from the American Thoracic Society, the American College of Chest Physicians, and the Pulmonary Vascular Research Institute to identify relevant studies.

## Results

The broad literature search strategy resulted in 2,646 potential observational studies in patients with Group 3 PH associated with COPD or ILD (Supplemental Table 1). The targeted title and abstract review identified 11 studies and 2 conference abstracts for claims-based studies focusing on Group 3 PH associated with COPD or ILD; 8 studies based in the US and 5 non-US (Table 1) (Supplemental Fig. 1).

**Table 1:** Administrative claims-based studies of group 3 PH associated with COPD or ILD in the literature

Reference	Data source	Study period	COPD or ILD identification	PH identification	Timing	Non-group 3 exclusions
Collard 2012	MarketScan Thomson Reuters: Commercial Claims and Encounters Database and the Medicare Supplemental and Coordination of Benefits Database	Jan 1, 2001–Sept 30, 2008	≥ 2 claims inpatient or outpatient claims on separate days associated with IPF (≥ 2 ICD-9-CM 516.3 **OR** at least 1 ICD-9-CM 516.3 and a subsequent ICD-9-CM 515 code) Excluded if the patient had ≥ 2 inpatient or outpatient claims with the same diagnosis code for another type of ILD on separate days	≥ 1 inpatient or outpatient claim of PH (ICD-9-CM: 416.0)	Incident PH was defined as PH diagnosis after the second lung disease code (PH after lung disease)	N/R
Collard 2015	5% random and representative sample of the US Medicare beneficiaries, Part A, and B files	2000–2011	≥ 1 outpatient or inpatient claim of IPF (ICD-9-CM 516.3) Excluded if there were ≥ 1 outpatient or inpatient diagnosis code for other ILD	≥ 1 inpatient or outpatient claim of PH (ICD-9-CM 416.0x)	Comorbid PH was determined in the pre-index period before IPF diagnosis (PH before lung disease)	N/R
Heresi 2017	Truven Health Analytics MarketScan Databases: Commercial Claims and Encounters Database and the Medicare Supplemental Database	July 1, 2010–June 30, 2013	≥ 1 claim for a lung disease associated with Group 3 PH	≥ 2 inpatient or outpatient claims for PH (ICD-9-CM 416.0 or 416.8) that were separated by at least 1 day but within 12 months of each other **AND** ≥ 1 claim for right heart catheterization or echocardiogram during the baseline period	Group 3 PH lung disease claim must have been during the baseline period before PH (PH after lung disease)	Patients with ≥ 1 claim with diagnosis codes or procedures related to Groups 2, 4, or 5 PH were excluded in the study period
Medrek 2017	Veterans Health Administration Corporate Data Warehouse (VISN 16 South Central)	2000–2012	≥ 1 hospitalization or ≥ 2 outpatient claims where COPD was the primary diagnosis (ICD-9-CM 491, 492, 494, 496)	≥ 1 outpatient or inpatient claim of PH (ICD-9-CM 416.0, 416.8)	Incident PH found in the post-index period after COPD (PH after lung disease)	N/R
Pedraza-Serrano 2019	Spanish National Hospital Discharge Database (95% of hospital discharges in Spain)	2014–2015	≥ 1 admission with ILD ICD-9-CM diagnosis codes: IPF (516.31), hypersensitivity pneumonitis (495.9), cryptogenic organizing pneumonia (516.36), lymphangioleiomyomatosis (516.4), pulmonary Langerhans cell histiocytosis (516.5), and sarcoidosis (135)	≥ 1 admission with a diagnosis code of PH (ICD-9-CM 416.0, 416.8)	COPD and PH diagnoses were found on the same claim (PH at same time with lung disease)	N/R
Kim 2018	Veterans Administration National Utilization and Pharmacy Data Systems	2005–2012	≥ 1 claim for a lung disease associated with Group 3 PH	≥ 1 inpatient or outpatient claim of PH (ICD-9-CM: 416.x) **AND** ≥ 1 prescription for daily PDE5i therapy. Daily is defined as pull per month ratio ≥ 30	Diagnosis code for PH must appear before the first daily PDE5i prescription	Patients were grouped into either Group 1, Group 4/5, or Group 2/3. For patients with diagnoses from multiple groups, an algorithm was used to assign PH groups, preferentially labeling patients as Group 1, 4, and 5 instead of Group 2/3,
Wijeratne 2018	Institute for Clinical Evaluative Sciences linked databases of universal healthcare coverage for Ontario, Canada residents along with the Ontario Drug Benefit database and the Canada Institute for Health Information databases	1993–2012	≥ 1 hospitalization or emergency department visit for a Group 3-related lung disease diagnosis code	≥ 1 hospitalization or emergency department visit for PH (ICD-9-CM 416.0, 416.1, 416.8, 416.9; ICD-10-CM I27.0, I27.1, I27.2, I27.8, I27.9)	Assessed for Group 3-related lung disease in the 5 years before the first PH claim (PH after lung disease)	≥ 1 claim for Groups 2 or 4 PH utilizing diagnosis codes. Patients with no Group 2, 3, or 4 PH diagnosis codes were assigned as Group 1. Patients could belong to multiple PH groups, with the exception of Group 1
Butt 2019	Danish Central Population Registry and the National Prescription Registry	1978–2015	≥ 1 outpatient or inpatient claim of first-time diagnosis of SSc (ICD-10-CM M34, except for M34.2)	≥ 1 outpatient or inpatient claim of PH (ICD-8-CM 426 or ICD-10-CM I27)	Incident PH was defined as PH diagnosis after SSc diagnosis (PH after lung disease)	N/R
Frank 2019	Scientific Institute of the Allgemeine Ortskrankenkasse Statutory Health Insurance Funds (WIdO) insurance claims	2009–2014	≥ 1 code for IPF (ICD-10-CM J84.1) or sarcoidosis (ICD-10-CM D86.0–D86.9) from outpatient or inpatient. Excluded any individuals without confirmed outpatient diagnoses from pulmonologist, an internal specialist, and without any inpatient diagnoses for the relevant diseases **AND** At least 1 relevant diagnostic procedure (bronchoscopy, lung computerized tomography, pulmonary function testing, or assessment of autoantibodies) from a visit with a relevant diagnosis	≥ 1 code for PH (ICD-10-CM 127.0, 127.8, 127.9) from inpatient or outpatient	Comorbid PH was determined in the same quarter as incident ILD diagnosis, either IPF or sarcoidosis (PH at the same time as lung disease)	N/R
Hemnes 2019	US PharMetrics Plus Commercial data set (pharmacy, medical, hospital claim; nationally representative)	2012–2016	≥ 2 claims from outpatient or inpatient claims ≥ 30 days apart for either COPD or ILD	≥ 2 claims from outpatient or inpatient claims ≥ 30 days apart for PH (ICD-9-CM 416.0, 416.8; ICD-10-CM I27.0, I27.20, I27.21, I27.23, I27.24, I27.29, I27.89)	COPD and ILD claims were required in the baseline period, prior to the PH claim (PH after lung disease)	Patients with ≥ 1 claim of Group 2, 4, or 5 PH in the pre-index period were excluded. Criteria included ICD-9/10-CM diagnosis, ICD-9/10-CM procedure, and CPT procedure codes
Trammell 2019	Veterans Health Administration Corporate Data Warehouse	Jan 2003–Sept 2015	≥ 1 outpatient or inpatient claim for a Group 3 PH-related lung disease diagnosis code	≥ 2 outpatient claims ≥ 30 days apart or ≥ 1 inpatient claim for PH (ICD-9-CM 416.0, 416.2, 416.8, 416.9)	Assessed for lung disease codes in the baseline period and up to 6 months after PH diagnosis (PH after and same time as lung disease)	≥ 1 claim of Group 2, 4, or 5 PH codes were assessed in baseline and up to 6 months after PH diagnoses If a patient did not have any Group 2, 3, 4, or 5 PH codes, they were counted as Group 1 If patients fell under multiple groups, then they were captured in the “multiple groups” cohort
Lautsch 2020	Optum’s Clinformatics Data Mart composed of commercial health plan data and Medicare Advantage members	2014–2018	≥ 2 outpatient or inpatient claims for COPD. Patients were excluded who had previous lung transplant, as determined by procedure codes	≥ 2 outpatient or inpatient claims for PH	COPD codes must have been prior to PH (PH after lung disease)	Excluded patients with ≥ 1 diagnosis of chronic thromboembolic PH or left heart disease PH and those with utilization of PAH-targeted therapy prior to the PH diagnosis
Wu 2020	Taiwan National Health Insurance plan database (includes hospitals, clinics, and pharmacies)	2002–2017	≥ 3 outpatient or ≥ 2 inpatient diagnosis claims for COPD (ICD-9-CM 490, 491, 492, 496) **AND** Treated using COPD medications (LABA, LABA/ICS, LAMA, LABA/LAMA, SABA, SAMA, SABA/SAMA, systemic beta-2-adrenoreceptor agonists, ICS, methylxanthines), according to outpatient claims for more than 28 days within 1 year after the primary COPD diagnosis Excluded patients with ≥ 1 claim from any source with connective tissue disease and sleep apnea (other Group 3 lung diseases)	≥ 3 outpatient claims or ≥ 2 inpatient claims or > 2 emergency room claims for PH (ICD-9-CM 416.0, 416.8, 416.9; ICD-10-CM I27.0, I27.2, I27.8, I27.9)	Patients with PH claims before COPD were excluded. Incident PH was assessed after COPD (PH after lung disease)	≥ 1 claim for ICD-9-CM diagnosis codes for select Group 1, 2, or 4 PH diseases

*COPD* chronic obstructive pulmonary disease, *CPT* Current Procedural Terminology, *ICD-8-/9-/10-CM* International Classification of Diseases, 8^th^ or 9^th^ or 10^th^ Revision, Clinical Modification, *ICS* inhaled corticosteroid, *ILD* interstitial lung disease, *IPF* idiopathic pulmonary fibrosis, *LABA* long-acting beta agonist, *LAMA* long-acting muscarinic antagonist, *N/R* not reported, *PAH* pulmonary arterial hypertension, *PDE5i* phosphodiesterase-5 inhibitor, *PH* pulmonary hypertension, *SABA* short-acting beta agonist, *SAMA* short-acting muscarinic antagonist, *SSc* systemic sclerosis, *US* United States

The focus of these studies varied. Four studies evaluated measures across multiple PH groups or Group 3 PH in general [16–19]. There were 3 studies on patients with COPD [20–22]: 2 studies each in ILD [23, 24] and IPF [25, 26] and 1 study in systemic sclerosis classified as Group 3 PH [27]. One study looked at PH in ILD, COPD, and combined ILD and COPD [28].

Most studies (*n* = 9) claimed to be identifying Group 3 PH and included steps in their methodology to filter for these patients (such as ensuring that PH occurred after COPD/ILD or excluded non-Group 3 PH) [16–22, 27, 28]. Other studies took additional steps in their methodology but did not specifically claim to identify Group 3 PH [26] or reported PH as a comorbidity to the lung disease [23–25].

In the following discussion section, we outline key components to consider in the development of an algorithm for Group 3 PH associated with COPD or ILD. For each, we outline the findings from the literature search that relate to the component followed by our summary and recommendations.

## Discussion

**Fig. 1:**
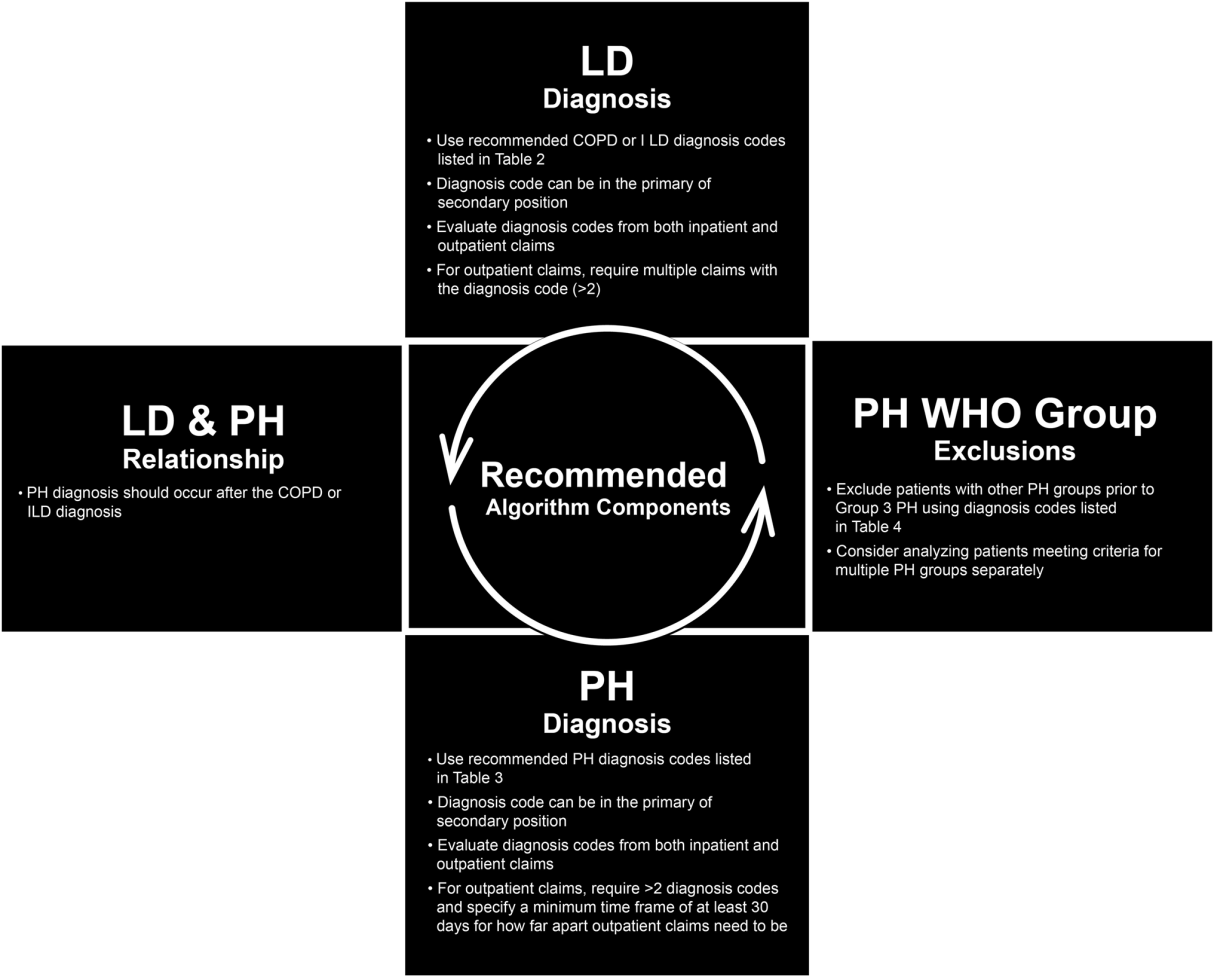
Recommended algorithm components – components of the recommended algorithm. COPD chronic obstructive pulmonary hypertension, ILD interstitial lung disease, LD lung disease, PH pulmonary hypertension

There are several components to consider when choosing an algorithm. The methods used in the published literature for identifying adult patients with Group 3 PH associated with COPD or ILD relied on the following considerations: (a) identification of COPD or ILD, (b) identification of PH, (c) defining the sequence between COPD/ILD and PH diagnoses, and (d) other PH groups or other Group 3 PH exclusions. We provide a summary of the published literature findings followed by considerations as to the impact of how restricting or relaxing the criteria for each of these components can impact the diagnostic performance of the algorithm. Components of the recommended algorithm are provided in Fig. 1.

### Identification of COPD or ILD

#### Findings in the Literature

The criteria used for the identification of COPD and ILD within the examined studies included (a) diagnosis codes, (b) relevant procedures, and (c) claims for medication. All 13 studies used diagnosis codes to identify patients with COPD or ILD, with 11 studies reporting ICD-9-CM codes [16–22, 24–26, 28] and 5 reporting ICD-10-CM codes [19, 20, 23, 27, 28].

Except for one COPD study conducted in the US that required the COPD diagnosis to be in the primary position [21], the remaining studies allowed the COPD *or* ILD diagnosis codes to be in either the primary or secondary position. Twelve studies identified patients from either inpatient or outpatient claims [16–23, 25–28] and the one remaining study used inpatient claims only [24]. In addition, 6 of the 12 studies using outpatient claims included a requirement for multiple claims (i.e., ≥ 2 claims) with a specified diagnosis [17, 20–22, 26, 28].

Only two studies utilized procedures specific to COPD or ILD to identify patients (i.e., pulmonary function tests in patients evaluated for ILD). Lautsch et al. excluded patients with prior lung transplant, while Frank et al. required patients to have at least one diagnostic procedure including bronchoscopy, lung computerized tomography (CT), pulmonary function testing, or assessment of autoantibodies [20, 23]. Two additional studies utilized procedures specific to COPD or ILD in sensitivity or subset analyses, including lung biopsy and CT [25, 26]. Requiring a procedure for COPD or ILD identification resulted in a significant drop in case count but did not change the general outcome of the studies.

#### Summary and Recommendations

After reviewing codes across studies, recommended diagnosis codes for COPD and ILD are provided in Table 2. While the US and non-US studies utilized similar ICD-9-CM codes for COPD and ILD, they differed more frequently when it came to ICD-10-CM. The US studies used codes down to the fifth digit, whereas non-US studies tended to use the higher, more general code down to the fourth digit. Thus, corresponding codes should be verified if international versions of ICD are used.

**Table 2:** Lung disease diagnosis codes for COPD and ILD

Lung disease subgroup	ICD-9-CM	ICD-10-CM	Description
Chronic obstructive pulmonary disease	491.0	J41.0	Simple chronic bronchitis
	491.1	J41.1	Mucopurulent chronic bronchitis
	491.8	J41.8	Other chronic bronchitis
	491.9	J42	Unspecified chronic bronchitis
	491.20		Obstructive chronic bronchitis without exacerbation
	492.0	J43.0	Unilateral pulmonary emphysema [MacLeod’s syndrome]
		J43.1	Panlobular emphysema
		J43.2	Centrilobular emphysema
	492.8	J43.8	Other emphysema
		J43.9	Emphysema, unspecified
	491.22	J44.0	Chronic obstructive pulmonary disease with (acute) lower respiratory infection
	491.21	J44.1	Chronic obstructive pulmonary disease with (acute) exacerbation
	496	J44.9	Chronic obstructive pulmonary disease, unspecified
Interstitial lung disease	517.1	J17	Rheumatic pneumonia
	500	J60	Coal worker’s pneumoconiosis
	501	J61	Pneumoconiosis due to asbestos and other mineral fibers
		J62.0	Pneumoconiosis due to talc dust
	502	J62.8	Pneumoconiosis due to other dust containing silica
	503	J63.0	Aluminosis (of lung)
		J63.1	Bauxite fibrosis (of lung)
		J63.2	Berylliosis
		J63.3	Graphite fibrosis (of lung)
		J63.4	Siderosis
		J63.5	Stannosis
		J63.6	Pneumoconiosis due to other specified inorganic dusts
	505	J64	Unspecified pneumoconiosis
		J65	Pneumoconiosis associated with tuberculosis
	504	J66	Airway disease due to specific organic dust
	495	J67	Hypersensitivity pneumonitis due to organic dust
	495.0	J67.0	Farmer’s lung
	495.1	J67.1	Bagassosis
	495.2	J67.2	Bird fancier’s lung
	495.3	J67.3	Suberosis
	495.4	J67.4	Malt worker’s lung
	495.5	J67.5	Mushroom worker’s lung
	495.6	J67.6	Maple-bark-stripper’s lung
	495.7	J67.7	Air conditioner and humidifier lung
	495.8	J67.8	Hypersensitivity pneumonitis due to other organic dusts
	495.9	J67.9	Hypersensitivity pneumonitis due to unspecified organic dust
	506	J68	Respiratory conditions due to inhalation of chemicals, gases, fumes, and vapors
	506.4	J68.4	Chronic respiratory conditions due to chemicals, gases, fumes, and vapors
	506.9	J68.9	Unspecified respiratory conditions due to chemicals, gases, fumes, and vapors
		J66.0	Byssinosis
		J66.1	Flax-dressers’ disease
		J66.2	Cannabinosis
		J66.8	Airway disease due to other specific organic dusts
	508.1	J70.1	Chronic and other pulmonary manifestations due to radiation
		J70.3	Chronic drug-induced interstitial lung disorders
		J70.4	Drug-induced interstitial lung disorders, unspecified
	508.8	J70.8	Respiratory conditions due to other specified external agents
	518.3	J82	Pulmonary eosinophilia
	516.2	J84.02	Pulmonary alveolar microlithiasis
	516.1	J84.03	Idiopathic pulmonary hemosiderosis
	515	J84.10	Pulmonary fibrosis, unspecified
	516.8	J84.11	Idiopathic interstitial pneumonia
	516.30	J84.111	Idiopathic interstitial pneumonia, NOS
	516.31	J84.112	Idiopathic pulmonary fibrosis
	516.32	J84.113	Idiopathic non-specific interstitial pneumonitis
	516.33	J84.114	Acute interstitial pneumonitis
	516.34	J84.115	Respiratory bronchiolitis interstitial lung disease
	516.36	J84.116	Cryptogenic organizing pneumonia
	516.37	J84.117	Desquamative interstitial pneumonia
		J84.17	Other interstitial pulmonary diseases with fibrosis, in diseases classified elsewhere
	516.35	J84.2	Lymphoid interstitial pneumonia
		J84.89	Other specified interstitial pulmonary disease
	516.9	J84.9	Interstitial pulmonary disease, unspecified
	710.0	M32.13	Lung involvement in systemic lupus erythematosus
	710.1	M34.81	Systemic sclerosis with lung involvement
	710.3	M33.01	Juvenile dermatomyositis with respiratory involvement
		M33.11	Other dermatomyositis with respiratory involvement
	710.4	M33.21	Polymyositis with respiratory involvement
		M33.91	Dermatopolymyositis, unspecified with respiratory involvement
	714.81	M05.1	Rheumatoid lung disease with rheumatoid arthritis
		M05.10	Rheumatoid lung disease with rheumatoid arthritis of unspecified site
		M05.11	Rheumatoid lung disease with rheumatoid arthritis of shoulder
		M05.111	Rheumatoid lung disease with rheumatoid arthritis of right shoulder
		M05.112	Rheumatoid lung disease with rheumatoid arthritis of left shoulder
		M05.119	Rheumatoid lung disease with rheumatoid arthritis of unspecified shoulder
		M05.12	Rheumatoid lung disease with rheumatoid arthritis of elbow
		M05.121	Rheumatoid lung disease with rheumatoid arthritis of right elbow
		M05.122	Rheumatoid lung disease with rheumatoid arthritis of left elbow
		M05.129	Rheumatoid lung disease with rheumatoid arthritis of unspecified elbow
		M05.13	Rheumatoid lung disease with rheumatoid arthritis of wrist
		M05.131	Rheumatoid lung disease with rheumatoid arthritis of right wrist
		M05.132	Rheumatoid lung disease with rheumatoid arthritis of left wrist
		M05.139	Rheumatoid lung disease with rheumatoid arthritis of unspecified wrist
		M05.14	Rheumatoid lung disease with rheumatoid arthritis of hand
		M05.141	Rheumatoid lung disease with rheumatoid arthritis of right hand
		M05.142	Rheumatoid lung disease with rheumatoid arthritis of left hand
		M05.149	Rheumatoid lung disease with rheumatoid arthritis of unspecified hand
		M05.15	Rheumatoid lung disease with rheumatoid arthritis of hip
		M05.151	Rheumatoid lung disease with rheumatoid arthritis of right hip
		M05.152	Rheumatoid lung disease with rheumatoid arthritis of left hip
		M05.159	Rheumatoid lung disease with rheumatoid arthritis of unspecified hip
		M05.16	Rheumatoid lung disease with rheumatoid arthritis of knee
		M05.161	Rheumatoid lung disease with rheumatoid arthritis of right knee
		M05.162	Rheumatoid lung disease with rheumatoid arthritis of left knee
		M05.169	Rheumatoid lung disease with rheumatoid arthritis of unspecified knee
		M05.17	Rheumatoid lung disease with rheumatoid arthritis of ankle and foot
		M05.171	Rheumatoid lung disease with rheumatoid arthritis of right ankle and foot
		M05.172	Rheumatoid lung disease with rheumatoid arthritis of left ankle and foot
		M05.179	Rheumatoid lung disease with rheumatoid arthritis of unspecified ankle and foot
		M05.19	Rheumatoid lung disease with rheumatoid arthritis of multiple sites

*ICD-9/10-CM* International Classification of Diseases, 9^th^ or 10^th^ Revision, Clinical Modification, *ILD* interstitial lung disease, *NOS* not otherwise specified

In addition, we recommend searching for COPD or ILD diagnoses in the primary or secondary position. When PH symptom exacerbations are due to underlying COPD or ILD, PH may be the primary reason for utilization diagnosis. We also recommend using both inpatient and outpatient claims unless doing so would affect study objectives (e.g., a study looking at hospital readmissions). When outpatient codes are used, we recommend using ≥ 2 to reduce the likelihood that a single diagnostic claim is used for patient identification.

We also note that PH is a complication of connective tissue disease and can be due to mechanisms other than ILD. Thus, caution should be considered with including connective tissue disease-related codes in the identification of ILD. We specifically recommend limiting diagnostic codes to connective tissue disease with respiratory or lung involvement. We also suggest alternative methods for the assessment of patients whose underlying conditions overlap across PH classification, such as analyzing patients meeting criteria for multiple PH groups separately.

Based on the findings from the evaluated studies, we do not recommend using any COPD/ILD-related procedure codes or medication claims for patient identification.

### Identification of PH

#### Findings in the Literature

The criteria used for the identification of PH within examined studies can be classified into the following: (a) diagnosis codes, (b) relevant procedures, (c) claims for medication, and (d) exclusions. All 13 studies used ICD-9-CM and ICD-10-CM diagnosis codes to identify patients with Group 3 PH associated with COPD or ILD. The majority (*n* = 11) [16–22, 24–26, 28] contained ICD-9-CM codes for patient identification, while six contained ICD-10-CM codes [19, 20, 22, 23, 27, 28]. No studies required the PH diagnosis to be in the primary position. Twelve studies identified patients from either inpatient or outpatient claims [16–23, 25–28] and the remaining one study used inpatient claims only [24]. The use of additional criteria such as a requirement for multiple claims was reported in five studies [16, 18, 20, 22, 28].

Only one study utilized PH-related procedures (i.e., right heart catheterization [RHC] or echocardiogram) in identification of patients with PH [16], and no studies required claims for any PH-related medications except Kim et al., which focused on appropriate use of phosphodiesterase-5 inhibitors (PDE5is) [17].

#### Summary and Recommendations

As all studies included PH-related diagnosis codes and there was general consensus on these codes across studies, we recommend utilizing a limited list of consensus codes (Table 3). Corresponding codes should be verified if international versions of the ICD are used. We recommend that PH diagnosis be in the primary or secondary position, as COPD or ILD associated with PH symptom exacerbation may be reported in the primary position. We also recommend using both inpatient and outpatient claims unless the study objectives are limited to one or the other. When outpatient codes are used, require at least two to reduce the likelihood that a single diagnostic claim is used for patient identification and specify a minimum time frame of at least 30 days for how far apart the outpatient claims need to be in order to qualify.

**Table 3:** PH diagnosis codes

ICD-9-CM	ICD-9-CM description	ICD-10-CM	ICD-10-CM description
416	Chronic pulmonary heart disease	I27	Other pulmonary heart diseases
416.0	Primary pulmonary hypertension	I27.0	Primary pulmonary hypertension
416.8	Other chronic pulmonary heart diseases	I27.2	Other pulmonary heart disease
		I27.20	Pulmonary hypertension, unspecified
		I27.21	Secondary pulmonary arterial hypertension
		I27.23	Pulmonary hypertension due to lung disease and hypoxia
		I27.29	Other secondary pulmonary hypertension
		I27.89	Other specified pulmonary heart diseases
416.9	Chronic pulmonary heart disease, unspecified	I27.81	Cor pulmonale (chronic)
		I27.9	Pulmonary heart disease, unspecified

*ICD-9/10-CM* International Classification of Diseases, 9^th^ or 10^th^ Revision, Clinical Modification, *PH* pulmonary hypertension

We generally do not suggest utilizing PH-related procedures. While the guideline-driven practice is to confirm cases by means of RHC, studies suggest that less than two-fifths of patients with PH have an RHC prior to diagnosis [16, 19] within 3 months before or after medication [29] or within 12 months following diagnosis [30]. We also note that claims data indicate if a patient received a diagnostic test but do not include test results, thus utilizing echocardiography, which is non-specific to Group 3 PH, would not improve the sensitivity of patient identification. Thus, we recommend limiting the use of procedure codes for sensitivity analysis in patient identification or to limit the study population when a purer cohort is required. Lastly, we do not suggest utilizing PH-related medications for patient identification, as therapies used in Group 1 PAH are often used on- and off-label for Group 3 PH associated with both COPD and ILD.

### The Sequence of the COPD/ILD and PH Diagnoses

#### Findings in the Literature

In order to confirm that COPD or ILD was a contributing factor to the development of PH, patients must have developed lung disease prior to PH. Eight of the 13 evaluated studies required the COPD or ILD diagnosis code to be prior to the PH diagnosis code [16, 19–22, 26–28], and one study required an underlying cause of PH (not limited to lung disease) to be documented prior to PDE5i prescription [17]. In fact, of the 8 studies that specifically claimed to be studying Group 3 PH, seven of them included this criterion [16, 19–22, 27, 28].

#### Summary and Recommendations

We recommend that PH diagnosis occur after the COPD or ILD diagnosis to align with the natural progression of this disease. Patients should be identified and indexed on their first claim with a PH diagnosis code and require a COPD or ILD diagnosis code in the baseline period at least six to 12 months prior to the PH diagnosis.

### Identification of Other PH Group and Other Group 3 PH Conditions

#### Findings in the Literature

When identifying patients with Group 3 PH, five studies used diagnosis codes to identify and exclude non-Group 3 PH patients [16, 17, 20, 22, 28] and two studies used diagnosis codes to separate patients who met criteria for multiple PH groups from those who met only Group 3 PH [18, 19]. In addition to diagnosis codes, two studies utilized claims for PAH-indicated medications [20] and two studies used procedure codes [16, 28] to exclude patients from Group 3 PH. One study used PDE5i guidelines to assign patients with multiple diagnoses to an “appropriate use” group [17]. Algorithm assignment across PH groups was compared to chart abstraction, resulting in a positive predictive value of 86% for possible inappropriate use across groups of PH.

#### Summary and Recommendations

We recommend methodology to exclude patients with other PH groups prior to their Group 3 PH using diagnosis codes provided in Table 4. We do not recommend excluding patients with other PH conditions that develop after their Group 3 PH diagnosis, as these are relatively uncommon occurrences and may arise from diagnostic workup rather than diagnostic confirmation, as claims data reflect clinical care provided rather than results. We acknowledge that approximately 34% of patients with PH have overlapping diagnoses [19] and that excluding these patients may bias results for some research objectives, and the combination of multiple comorbidities may render patients vulnerable to developing PH and contribute to poor prognosis [19]. Thus, an alternative method would be to analyze patients meeting criteria for multiple PH groups separately or assign patients to a group based on the objectives of the study [17].

**Table 4:** Other exclusionary PH diagnosis codes

	ICD-9-CM	ICD-9-CM description	ICD-10-CM	ICD-10-CM description
Group 1: PAH				
Drug and toxin induced	995.29	Unspecified adverse effect of other drug medicinal and biological substance	T50.5X50	Adverse effect of appetite depressant
Associated with other systemic diseases	042	HIV	B20	HIV
	572.3	Portal hypertension	K76.6	Portal hypertension
	745.5	Atrial septal defect	Q21.1	Atrial septal defect
	745.4	Ventricular septal defect	Q21.0	Ventricular septal defect
	120.x	Schistosomiasis	B65.x	Schistosomiasis
Group 2: PH with left heart disease				
Left ventricular systolic dysfunction	414.10	Aneurysm of heart (wall)	I25.3	Aneurysm of heart
			I27.22	Pulmonary hypertension due to left heart disease
Left ventricular diastolic dysfunction	425.3	Endocardial fibroelastosis	I42.4	Endocardial fibroelastosis
	428.1	Left heart failure	I50.1	Left ventricular failure
	428.2	Systolic heart failure	I50.22	Chronic systolic heart failure
	428.3	Diastolic heart failure	I50.3	Diastolic heart failure
Valvular disease	394, 424.0	Disease/disorders of mitral valve	I34.0, I34.8	Non-rheumatic mitral valve insufficiency/disorder
	395, 424.1	Disease/disorders of aortic valve	I35.x	Disease/disorders of aortic valve
	396	Disease of mitral and aortic valve	I08	Disease of mitral and aortic valve
	746.3–746.7, 746.81	Mitral/aortic valve surgery	Q23	Mitral/aortic valve surgery
Congenital/acquired left heart inflow/outflow tract obstruction and congenital cardiomyopathies	425.1	Hypertrophic obstructive cardiomyopathy	I42.1	Hypertrophic obstructive cardiomyopathy
	425.8	Cardiomyopathy in other diseases classified elsewhere	I43	Cardiomyopathy in other diseases classified elsewhere
	746.8	Other specified congenital anomalies of heart	Q24	Other specified congenital anomalies of heart
Hypertensive heart disease	402.01, 402.11, 402.91	Malignant hypertensive heart disease with heart failure	I11.0	Hypertensive heart disease with heart failure
	402.11	Benign hypertensive heart disease with heart failure		
	402.91	Unspecified hypertensive heart disease with heart failure		
Hypertensive heart and kidney disease	404.01, 404.03, 404.11, 404.13, 404.91, 404.93	Hypertensive heart and chronic kidney disease with heart failure and chronic kidney disease stage 1–4 or unspecified	I13.0, I13.2	Hypertensive heart and chronic kidney disease with heart failure and stage 1–4 chronic kidney disease or unspecified
	404.03, 404.13, 404.93	Hypertensive heart and chronic kidney disease with heart failure and with chronic kidney disease stage 5 or end-stage renal disease	I13.2	Hypertensive heart and chronic kidney disease with heart failure and with stage 5 chronic kidney disease or end-stage renal disease
Group 3: Other lung diseases				
Alveolar hypoventilation disorder	327.24	Idiopathic sleep-related non-obstructive alveolar hypoventilation	G47.34	Idiopathic sleep-related non-obstructive alveolar hypoventilation
	327.25	Congenital central alveolar hypoventilation syndrome	G47.35	Congenital central alveolar hypoventilation syndrome
Chronic exposure high altitude	E902.0	Accident due to residence or prolonged visit at high altitude	W94.11XA	Exposure to residence or prolonged visit at high altitude, initial encounter
			W94.11XD	Exposure to residence or prolonged visit at high altitude, subsequent encounter
			W94.11XS	Exposure to residence or prolonged visit at high altitude, sequela
	993.2	Other and unspecified effects of high altitude		
Developmental lung diseases	519.2	Mediastinitis	J98.15	Mediastinitis
	756.6	Anomalies of diaphragm	Q79.0	Congenital diaphragmatic hernia
	770.7	Chronic respiratory disease arising in the perinatal period	P27.1	Bronchopulmonary dysplasia originating in the perinatal period
	516.64	Alveolar capillary dysplasia with vein misalignment	J84.843	Alveolar capillary dysplasia with vein misalignment
	748.5	Agenesis, hypoplasia, and dysplasia of lung	Q33.3 Q33.6	Agenesis of lung Congenital hypoplasia and dysplasia of lung
	516.63	Surfactant mutations of the lung	J84.83	Surfactant mutations of the lung
	516.62	Pulmonary interstitial glycogenosis	J84.842	Pulmonary interstitial glycogenosis
	516.0	Pulmonary alveolar proteinosis	J84.01	Alveolar proteinosis
	516.69	Other ILD of childhood	J84.848	Other ILD of childhood
Group 4: CTEPH				
CTEPH	415.1	Pulmonary embolism	I26.99	Other pulmonary embolism
			I26.90	Septic pulmonary embolism without acute cor pulmonale
	416.2	Chronic pulmonary embolism	I27.24	Chronic thromboembolic pulmonary hypertension
			I27.82	Chronic pulmonary embolism
	V12.51	History of venous thrombosis and embolism	Z86.718	Personal history of other venous thrombosis and embolism
Group 5: PH with unclear multifactorial mechanisms				
Hematologic disorders	282	Hereditary hemolytic anemias	D55	Anemia due to enzyme disorders
	283	Acquired hemolytic anemias	D56	Thalassemia
	283.4	Polycythemia vera	D57	Sickle cell disorders
	238.79	Other lymphatic and hematopoietic tissues	D58	Other hereditary hemolytic anemias
Systemic disorders	135	Sarcoidosis	D86	Sarcoidosis
	277.89	Other specified disorders of metabolism	E88.89	Other specified metabolic disorders
	202.5	Letterer–Siwe disease	C96.0	Multifocal and multisystemic (disseminated) Langerhans cell histiocytosis
	228.1	Lymphangioma any site	D18.1	Lymphangioma, any site
Metabolic disorders	271	Disorders of carbohydrate transport and metabolism	E74	Other disorders of carbohydrate metabolism
	272.7	Gaucher disease	E75.22	Gaucher disease
Others	519.2	Mediastinitis	J98.51	Mediastinitis
	746	Other congenital anomalies of the heart	Q22	Congenital malformations of pulmonary and tricuspid valves
			Q23	Congenital malformations of aortic and mitral valves
			Q24	Other congenital malformations of heart

*CTEPH* chronic thromboembolic pulmonary hypertension, *ICD-9/10-CM* International Classification of Diseases, 9^th^ or 10^th^ Revision, Clinical Modification, *PH* pulmonary hypertension

Using diagnosis codes in the identification of Group 3 PH does have some limitations. ICD-9-CM codes do not have specificity of classification for secondary PH, and it was not until ICD-10-CM that diagnosis codes provided for greater clinical classification of secondary PH. In addition, diagnosis codes do not reflect severity of the disease, particularly as they relate to distinguishing between multiple underlying causes of disease. So, for a patient with mild heart disease but severe lung disease that contributes significantly to PH, using real-world claims data may inadvertently misclassify this person as Group 2 PH.

In addition, there are some notable considerations. Given the overlap of secondary PH-related conditions in Group 1 PAH and Group 3 PH, we do not recommend using I27.x codes when developing exclusion criteria. Further, when conducting a study to identify Group 3 PH with COPD only, we recommend all patients with diagnosis codes for connective tissue disease be excluded. Lastly, given the high prevalence of sleep disorder breathing in COPD and ILD, we do not recommend excluding patients with this diagnosis.

None of the examined studies utilized PH-related medications to directly identify Group 3 PH, but one study did exclude patients with evidence of PAH-indicated medications [20].

As inhaled treprostinil is now FDA approved in both Group 1 PAH and Group 3 PH, and it is likely that medications approved for use in Group 1 PAH are being used off-label in Group 3 PH to improve exercise capacity, we do not recommend relying on medication use to identify or exclude patients.

## Conclusion

Correctly identifying adult patients with Group 3 PH associated with COPD or ILD in claims-based studies can improve the value of research findings for application in clinical care and population health, the utility of real-world evidence in support of FDA regulatory approvals, and more accurately inform formulary decision-making. When using the recommendations provided herein, care should be taken to consider policy and regulatory changes, such as FDA approvals and updated guidelines and their impact on how patients are identified. While some studies reported that they used validated codes or algorithms for either COPD/ILD or PH, it is important to note that only one reviewed study provided results on the validation of their patient identification algorithm. Future research should be conducted to validate patient identification algorithms, especially the combination of criteria required for the identification of Group 3 PH associated with COPD or ILD.

## Supplementary Information

Below is the link to the electronic supplementary material.

Supplementary file1 (PPTX 44 kb)

Supplementary file2 (DOCX 17 kb)

## Data Availability

All data generated or analyzed during this study are included in this published article or the supplementary information files.
