# Supplementary material for: Identifying Patients with Group 3 Pulmonary Hypertension Associated with COPD or ILD Using an Administrative Claims Database
Source: Lung. 2022 Mar 29;200(2):187–203. doi: 10.1007/s00408-022-00521-6 (PMC9038884; doi:10.1007/s00408-022-00521-6)
Supplement: Supplementary file 1 — Supplementary file1 (PPTX 44 kb) [file 408_2022_521_MOESM1_ESM.pptx]

## Slide 1
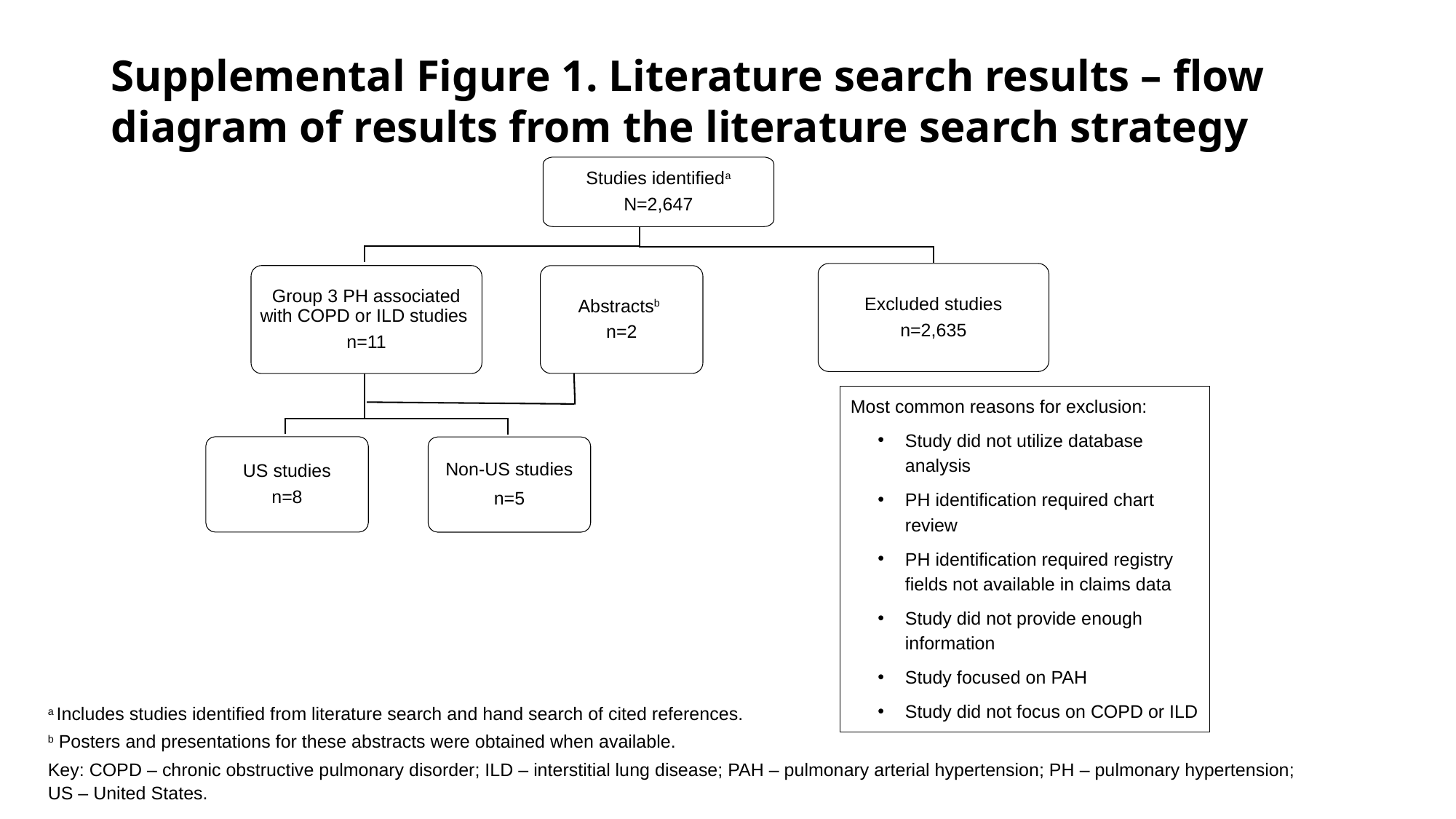

Supplemental Figure 1. Literature search results – flow diagram of results from the literature search strategy
Studies identifieda
N=2,647
Excluded studies
n=2,635
Group 3 PH associated with COPD or ILD studies
n=11
Abstractsb
n=2
Most common reasons for exclusion:
Study did not utilize database analysis
PH identification required chart review
PH identification required registry fields not available in claims data
Study did not provide enough information
Study focused on PAH
Study did not focus on COPD or ILD
US studies
n=8
Non-US studies
n=5
a Includes studies identified from literature search and hand search of cited references.
b Posters and presentations for these abstracts were obtained when available.
Key: COPD – chronic obstructive pulmonary disorder; ILD – interstitial lung disease; PAH – pulmonary arterial hypertension; PH – pulmonary hypertension; US – United States.
