# Supplementary material for: Identifying Patients with Group 3 Pulmonary Hypertension Associated with COPD or ILD Using an Administrative Claims Database
Source: Lung. 2022 Mar 29;200(2):187–203. doi: 10.1007/s00408-022-00521-6 (PMC9038884; doi:10.1007/s00408-022-00521-6)
Supplement: Supplementary file 2 — Supplementary file2 (DOCX 17 kb) [file 408_2022_521_MOESM2_ESM.docx]

Supplemental Table 1. Search strategy

| **Search** | **Focus of search** | **Search terms** |
| --- | --- | --- |
| #1 | Lung disease and/or hypoxia | ‘interstitial lung disease’ OR ‘ILD’ OR ‘idiopathic pulmonary fibrosis’ OR ‘pulmonary fibrosis‘ OR ‘lung fibrosis’ OR ’IPF’ OR ‘lung disease’ OR ‘chronic lung disease’ OR ‘CLD’ OR ‘chronic obstructive pulmonary disease’ OR ‘COPD’ OR ‘cystic fibrosis’ OR ‘CF’ OR ‘lung dysplasia’ OR ‘bronchopulmonary dysplasia’ OR ‘idiopathic interstitial pneumonia’ OR ‘emphysema’ OR ‘combined pulmonary fibrosis and emphysema’ OR ‘CPFE’ OR ‘lymphangioleiomyomatosis’ OR ‘hypersensitivity pneumonitis’ OR ‘allergic pneumonitis’ OR ‘interstitial pneumonitis’ OR ‘parenchymal disease’ OR ‘diffuse parenchymal lung disease’ OR ‘DPLD’ OR ‘kyphoscoliosis’ OR ‘kyfoscoliosis’ OR ‘obesity-hypoventilation syndrome’ OR ‘systemic sclerosis’ OR ‘pneumoconiosis’ OR ‘pneumokoniosis’ |
| #2 | Pulmonary hypertension | ‘pulmonary hypertension’ OR ‘PH’ OR ‘pulmonary hypertensive’ OR ‘pulmonary’ NEXT/2 ‘hypertension’ OR ‘lung’ NEXT/2 ‘hypertension’ |
| #3 | Group 3 PH | ‘COPD-PH’ OR ‘PH-COPD’ OR ‘CLD-PH’ OR ‘PH-CLD’ OR I27.2* OR ‘Group 3 PH’ OR ‘PH group 3’ OR ‘PH WHO Group 3’ |
| #4 | Retrospective study design terms and publication types | ‘retrospective’ OR ‘retrospectively’ OR ‘database’ OR ‘cohort’ OR ‘claims’ OR ‘database’ OR ‘registry’ OR ‘registries’ OR ‘observational’ OR ‘real world’ OR ‘electronic medical’ OR ‘EMR’ OR ‘cohort analysis’ |
| #5 | Meta analyses or literature reviews | ‘meta analysis’ OR ‘meta analyses’ OR ‘systematic review’ OR ‘meta-analysis’ OR ‘meta-analyses’ |
| #6 | Healthcare management-related studies | ‘health care management’ |
| #7 | Final articles for title and abstract review | ([#1 AND #2] OR #3) AND (#4 OR #5 OR #6) |

Note: /exp and /syn commands were utilized as appropriate to improve search criteria results. Search terms were applied to title, abstract, and index terms. All studies were limited to humans, English language, and indexed 2010 through June 2020.

Note: Search criteria focused on Group 3 PH, thus identifying a larger set of potential articles. Additional inclusion and exclusion criteria were applied to identify articles focusing on the COPD and ILD patient population.

Key: CF – cystic fibrosis; CLD – chronic lung disease; COPD – chronic obstructive pulmonary disease; CPFE – combined pulmonary fibrosis and emphysema; DPLD – diffuse parenchymal lung disease; ILD – interstitial lung disease; IPF – idiopathic pulmonary fibrosis; PH – pulmonary hypertension.
